# Supplementary material for: Antiproliferative Imidazo-Pyrazole-Based Hydrogel: A Promising Approach for the Development of New Treatments for PLX-Resistant Melanoma
Source: Pharmaceutics. 2023 Oct 4;15(10):2425. doi: 10.3390/pharmaceutics15102425 (PMC10610107; doi:10.3390/pharmaceutics15102425)
Supplement: Supplementary file 1 [file pharmaceutics-15-02425-s001.zip › pharmaceutics-2597673-supplementary.pdf]

pharmaceutics

MDPI

Supplementary Materials

Antiproliferative Imidazo-Pyrazole-Based Hydrogel: A Promising Approach for the Development of New Treatments for PLX-Resistant Melanoma

Silvana Alfei [1,*], Marco Milanese [1], Chiara Brullo [2], Giulia Elda Valenti [3], Cinzia Domenicotti [3], Eleonora Russo [2], and Barbara Marengo [3,*]

[1] Section of Chemistry and Pharmaceutical and Food Technologies, Department of Pharmacy, University of Genoa, Viale Cembrano, 4, 16148 Genoa, Italy; marco.milanese@unige.it
[2] Section of Medicinal Chemistry and Cosmetic Product, Department of Pharmacy (DIFAR), University of Genoa, Viale Benedetto XV, 3, 16132 Genoa, Italy; chiara.brullo@unige.it (C.B.); eleonora.russo@unige.it (E.R.)
[3] Department of Experimental Medicine (DIMES), University of Genova, Via Alberti L.B., 16132 Genoa, Italy; giuliaelda.valenti@edu.unige.it (G.E.V.); cinzia.domenicotti@unige.it (C.D.)
* Correspondence: alfei@difar.unige.it (S.A.); barbara.marengo@unige.it (B.M.); Tel.: +39-010-355-2296 (S.A.)

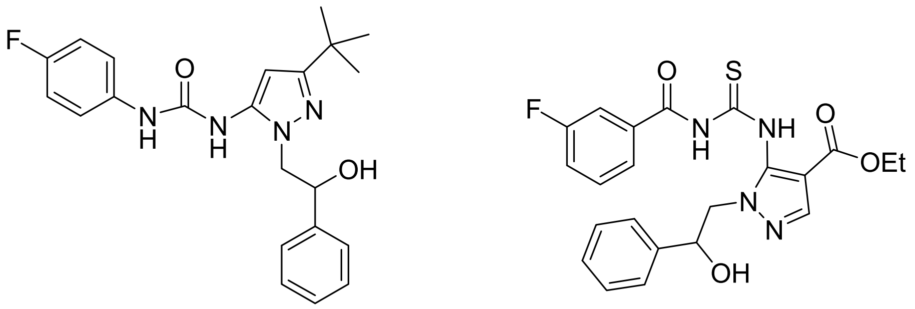

a **b**

Figure S1. Chemical structure of previously reported antìbacterial pyrazoles **3c** (**a**) and **4b** (**b**).

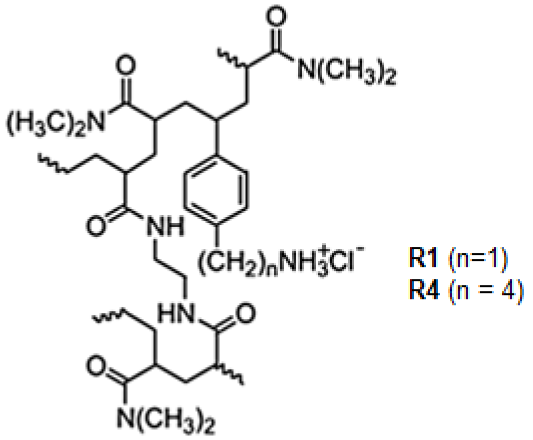

Figure S2. Chemical structure of previously reported resin R1 (n = 1) and R4 (n = 4).

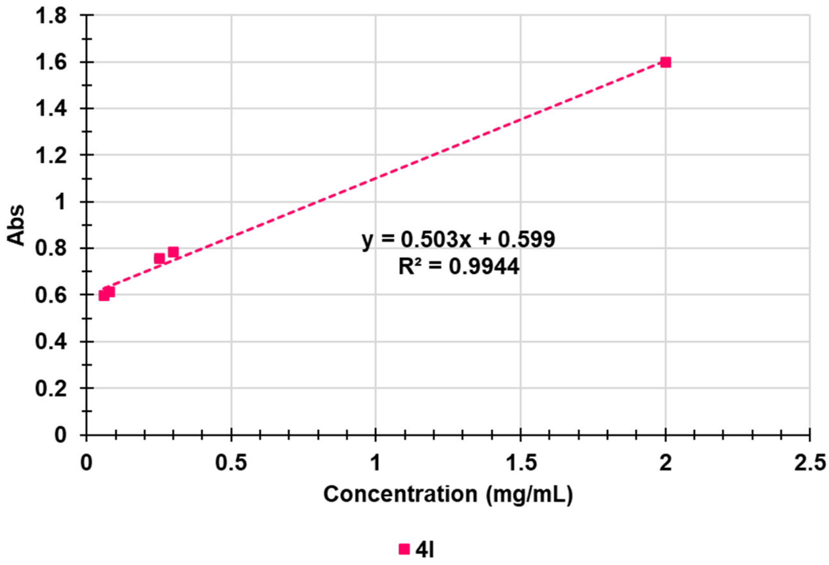

Figure S3. 4I calibration curve.

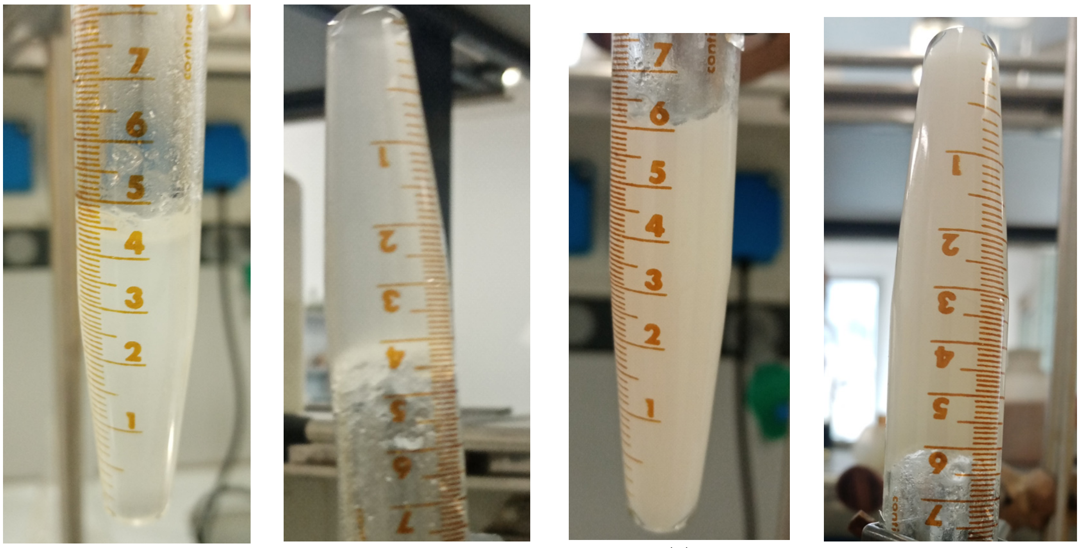

(a) (b) (c) (d)

Figure S4. Appearance of R4HG (**a**,**b**) and R4HG-4I (**c**,**d**) at their equilibrium degree of swelling (EDS).

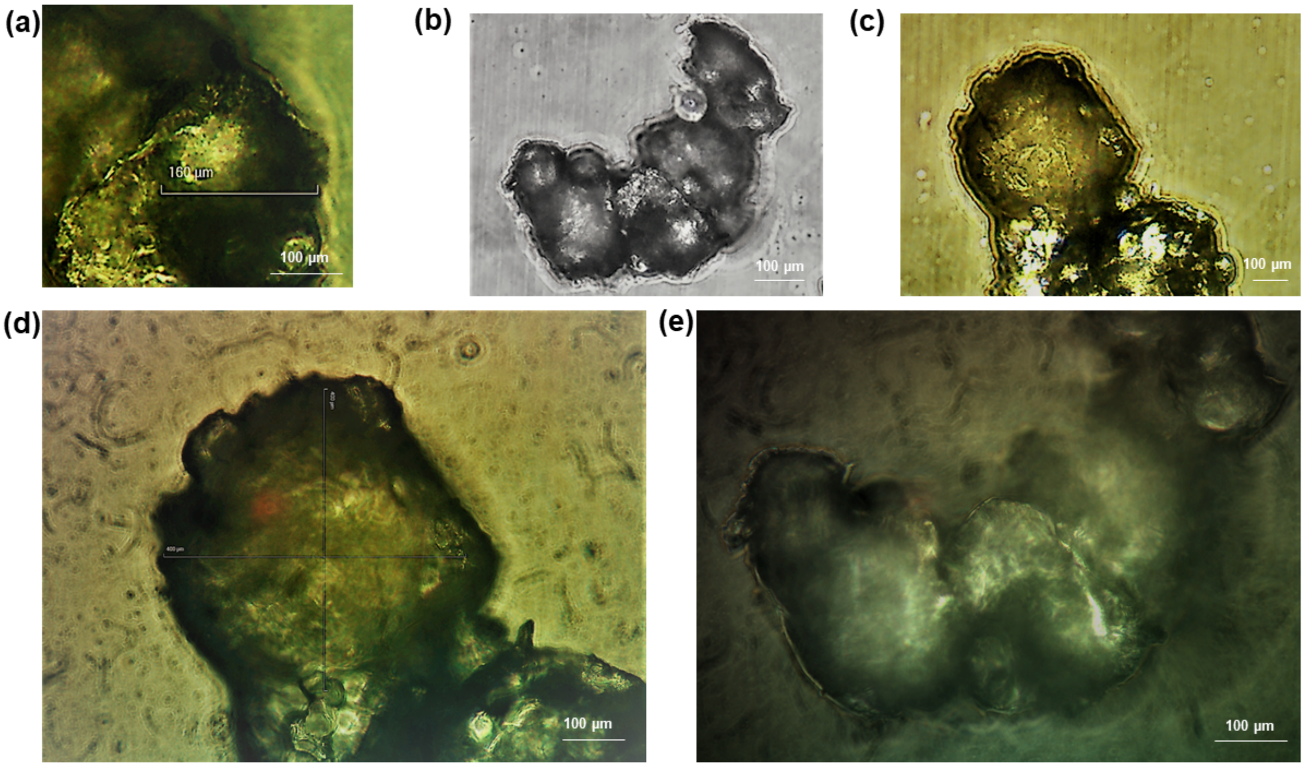

Figure S5. Representative optical microphotographs of R4HG after drying at 100°C util a constant weight equal to that before swelling was achieved (R4HG-D). Objective 40 × (**a**, **d**, **e**), 20 × (**b**, **c**).

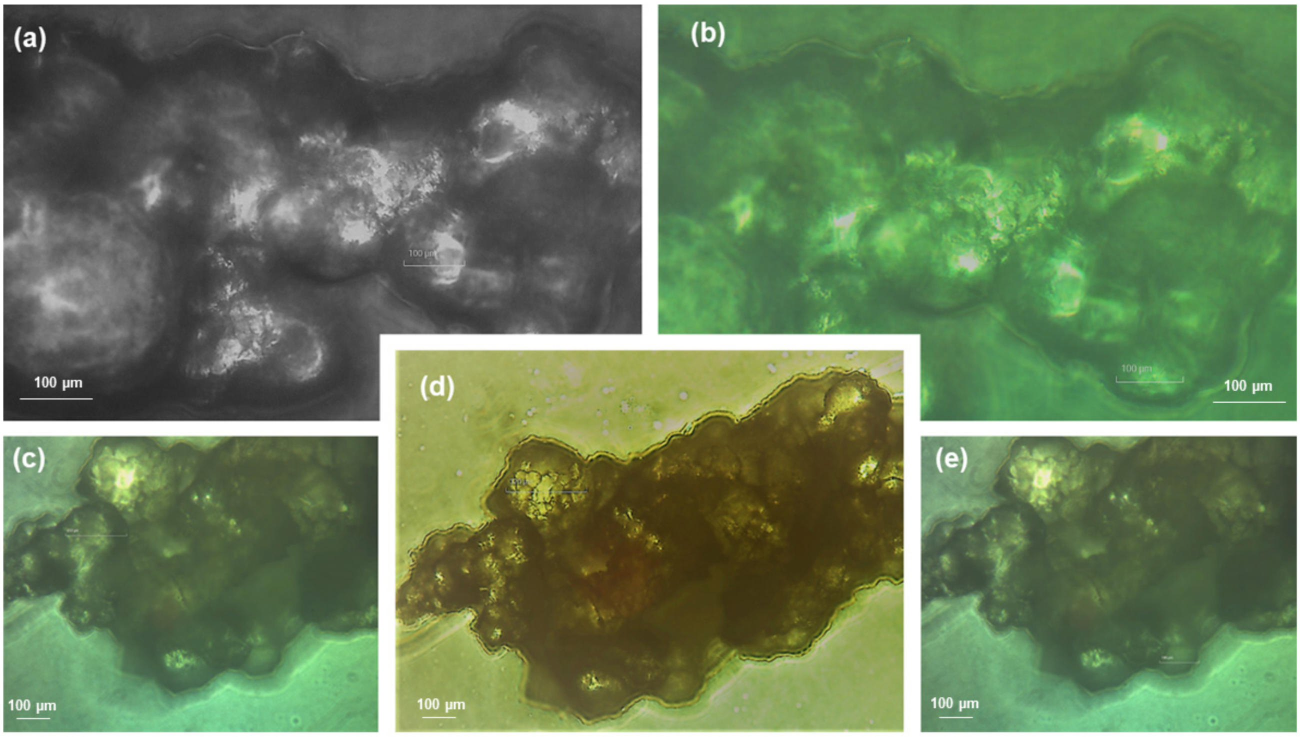

Figure S6. Representative optical microphotographs of R4HG-4I after drying at 100°C util a constant weight equal to that before swelling was achieved (R4HG-4I-D). Objective 40 × (**a**, **b**), 20 × (**c**, **d**, **e**).

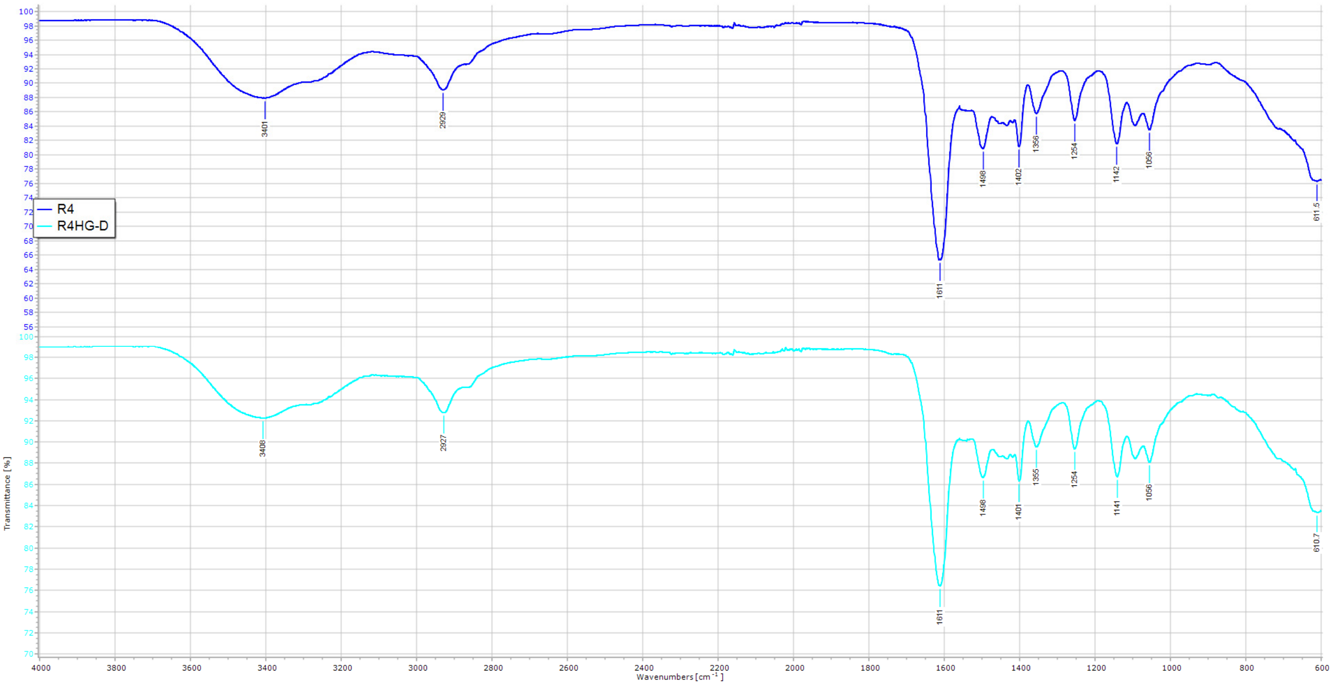

Figure S7. Representative ATR-FTIR spectra of resin R4 and fully dried hydrogel R4HG-D.

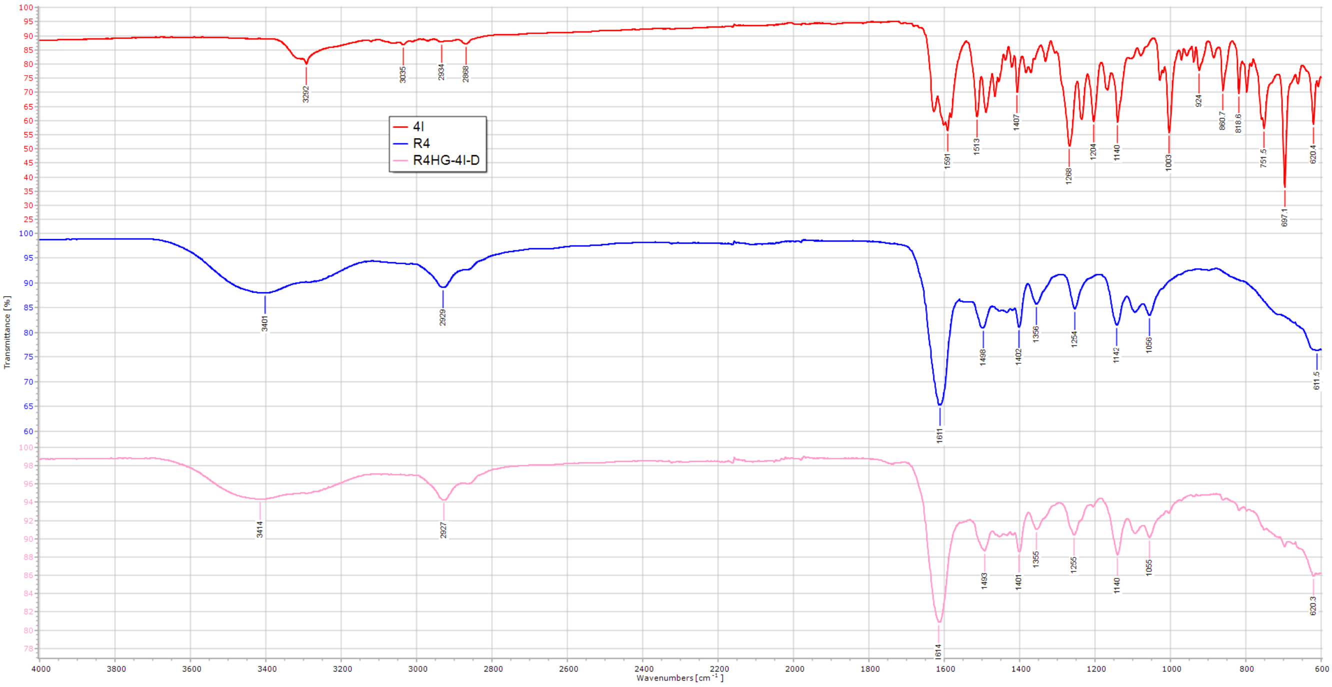

Figure S8. Representative ATR-FTIR spectra of resin R4, **4I** and fully dried hydrogel R4HG-4I-D.

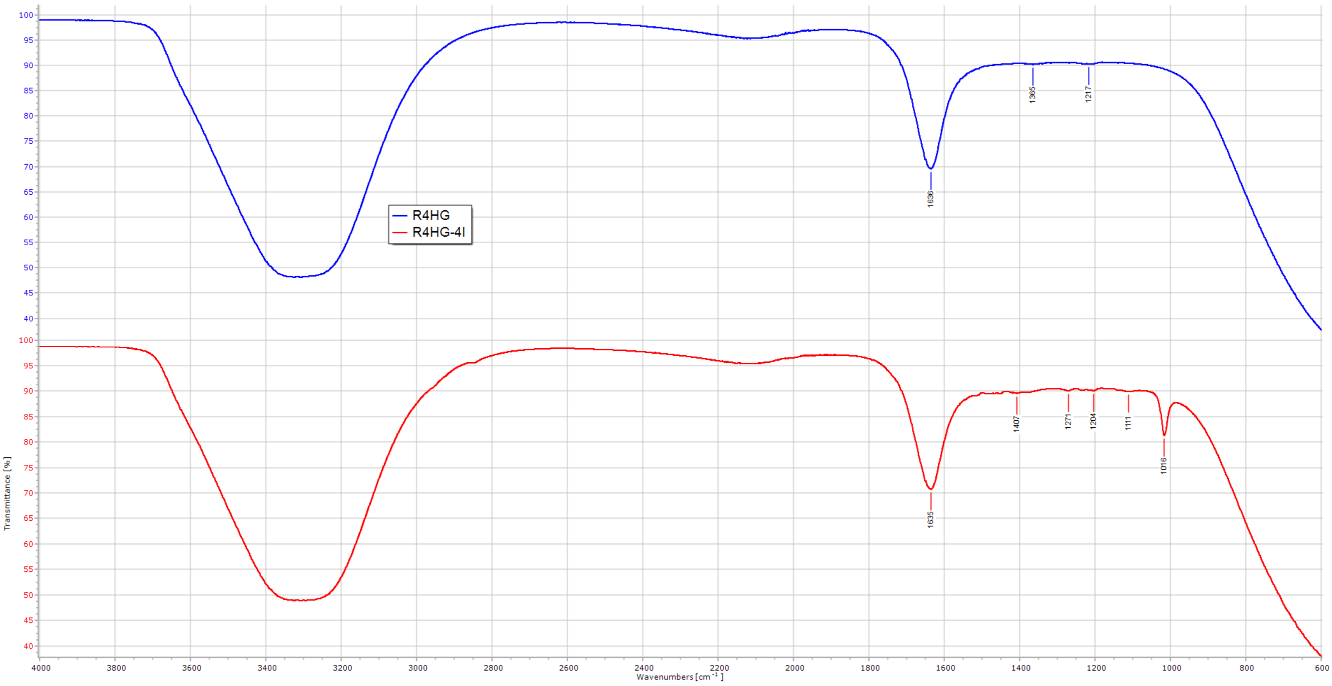

Figure S9. Representative ATR-FTIR spectra of swollen hydrogels R4HG and R4HG-4I.

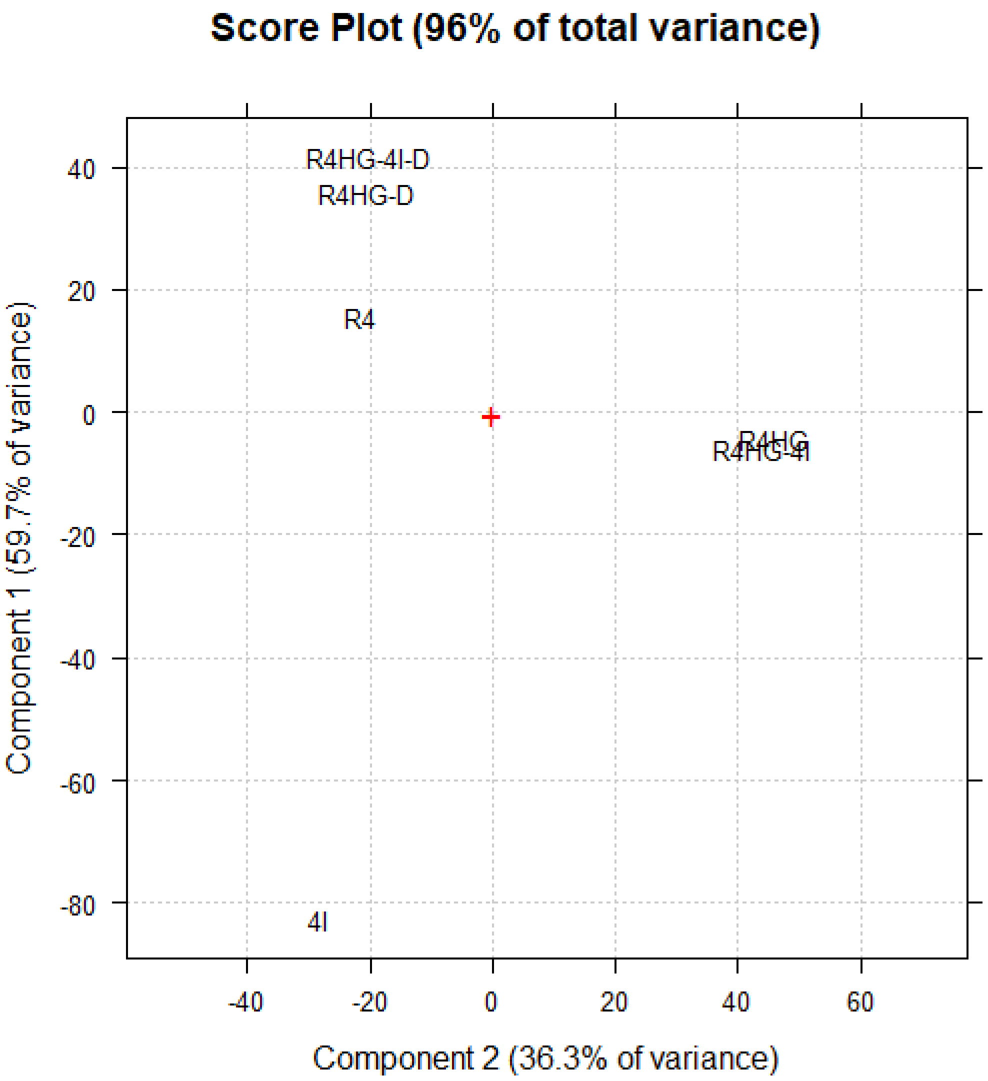

Figure S10. Score plot of PC1 (explaining the 59.7% of variance) vs. PC2 (explaining the 36.3% of variance).

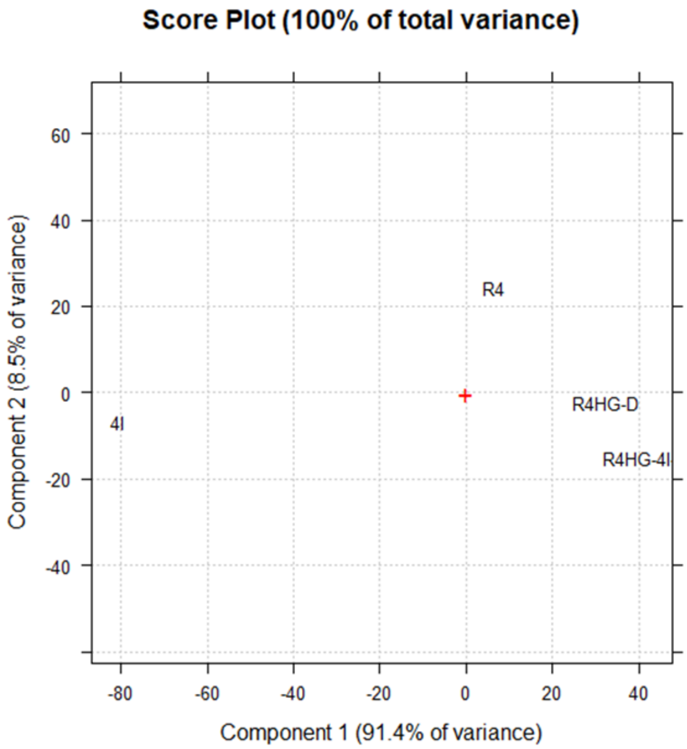

Figure S11. Score plot of PC1 (explaining the 91.4% of variance) vs. PC2 (explaining the 8.5% of variance).

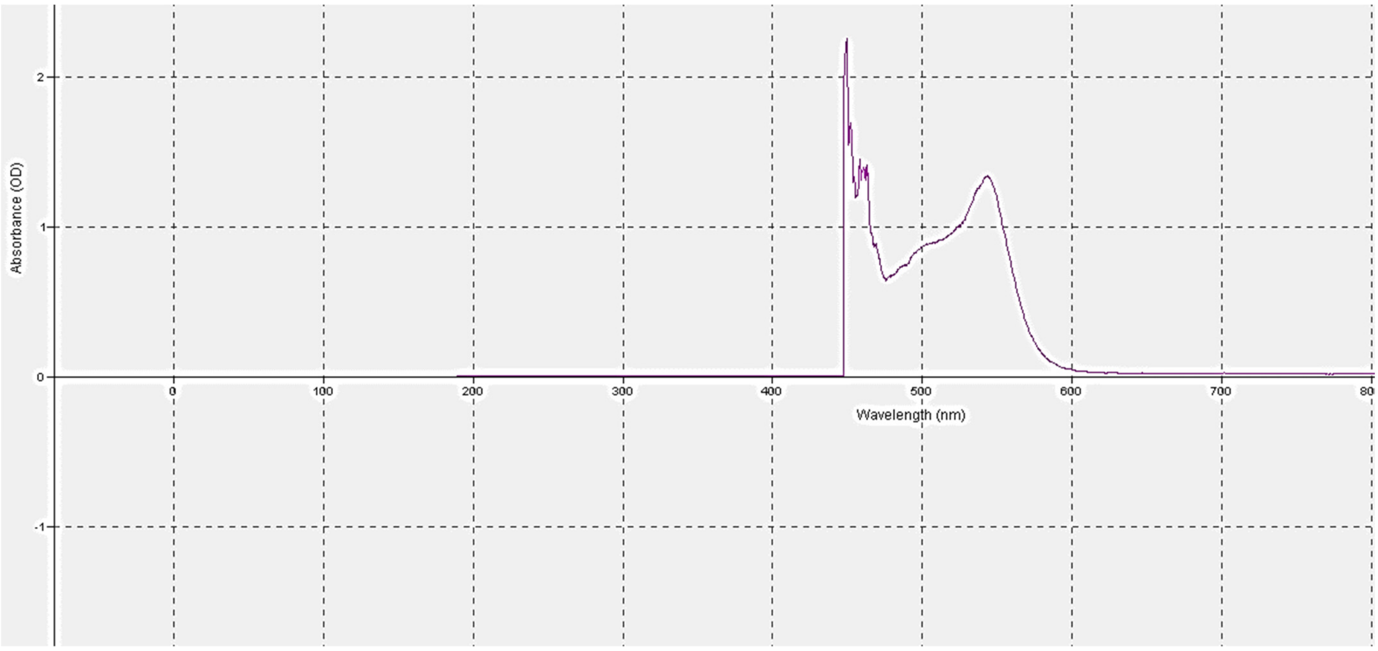

Figure S12. UV-Vis spectrum of **4I**.

Video S1. https://clipchamp.com/watch/LVAKVdP1F8f.

Video S2. https://clipchamp.com/watch/TtDuSbTIuWF.

Table S1. Values of the coefficients of determination obtained for all the kinetic models used.

Kinetic Model	R^2 of R4HG	R^2 of R4HG-4I
Zero-order	0.8940	0.8451
First-order	0.9594	0.9422
Hixson–Crowel	0.8940	0.8451
Higuchi	0.9684	0.9580
**Korsmeyer–Peppas**	**0.9828**	**0.9711**

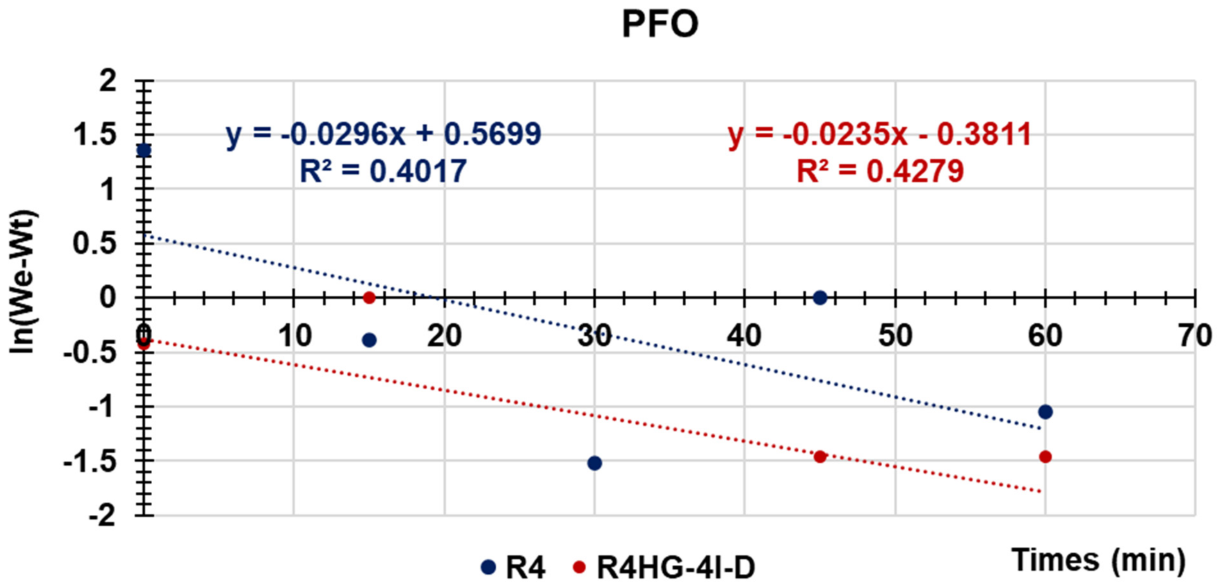

Figure S13. PFO kinetic model (We and Wt expressed as mg).

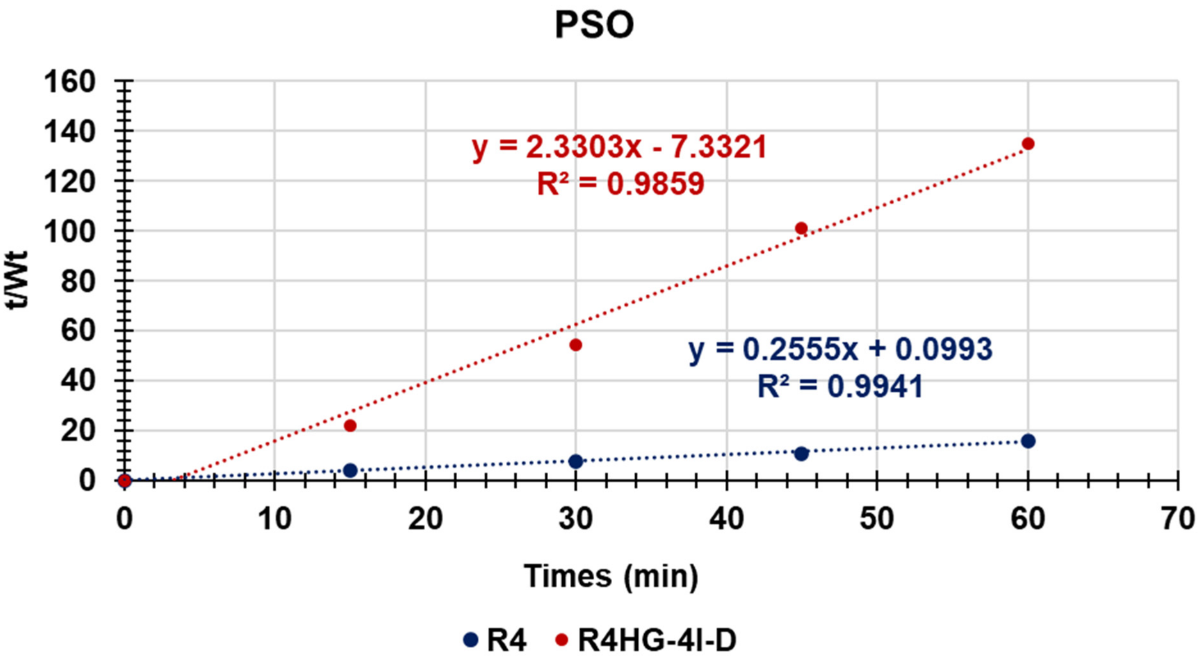

Figure S14. PSO kinetic model (Wt expressed as mg; t/Wt = mg × min).

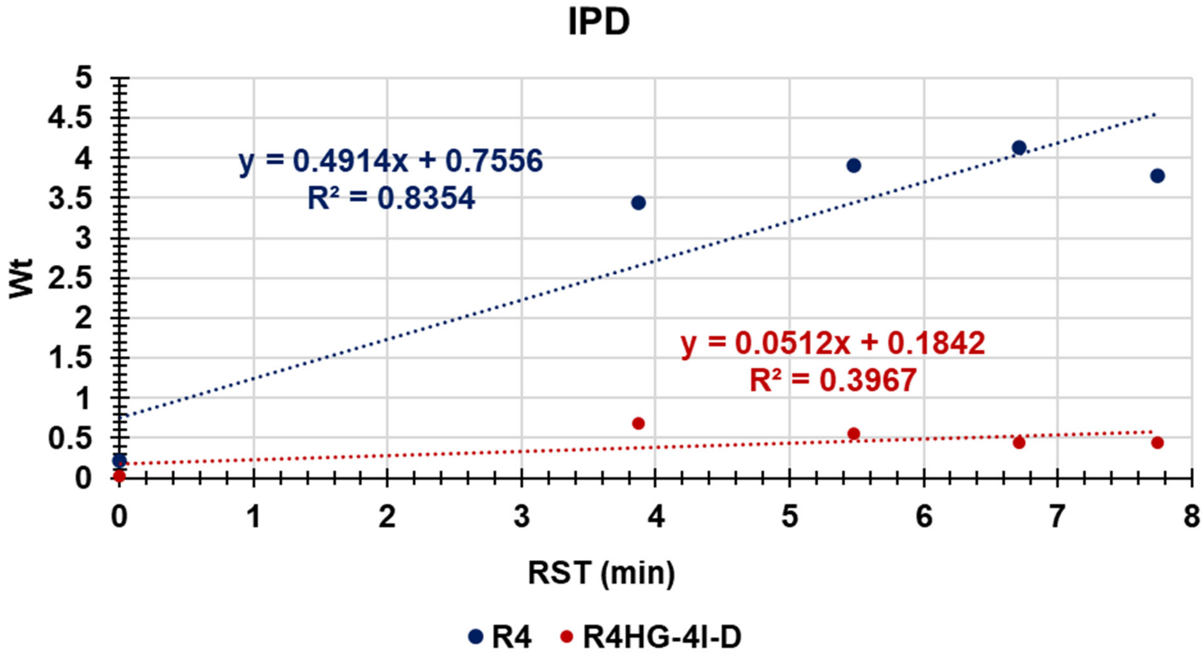

Figure S15. IPD kinetic model (Wt expressed as mg). RST = root square of times.

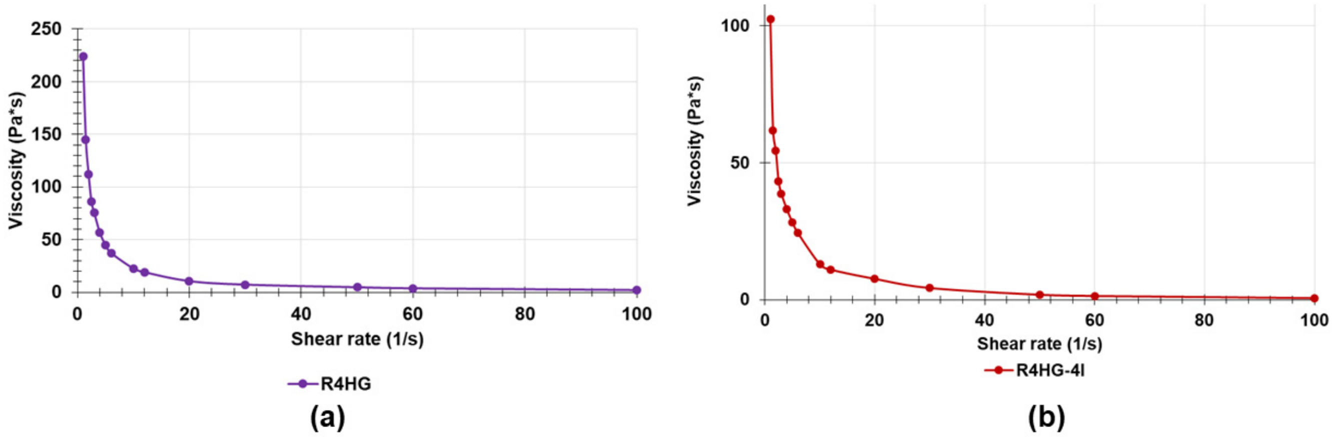

Figure S16. Curve of the viscosity vs. shear rate of R4HG (**a**) and R4HG-4I (**b**).

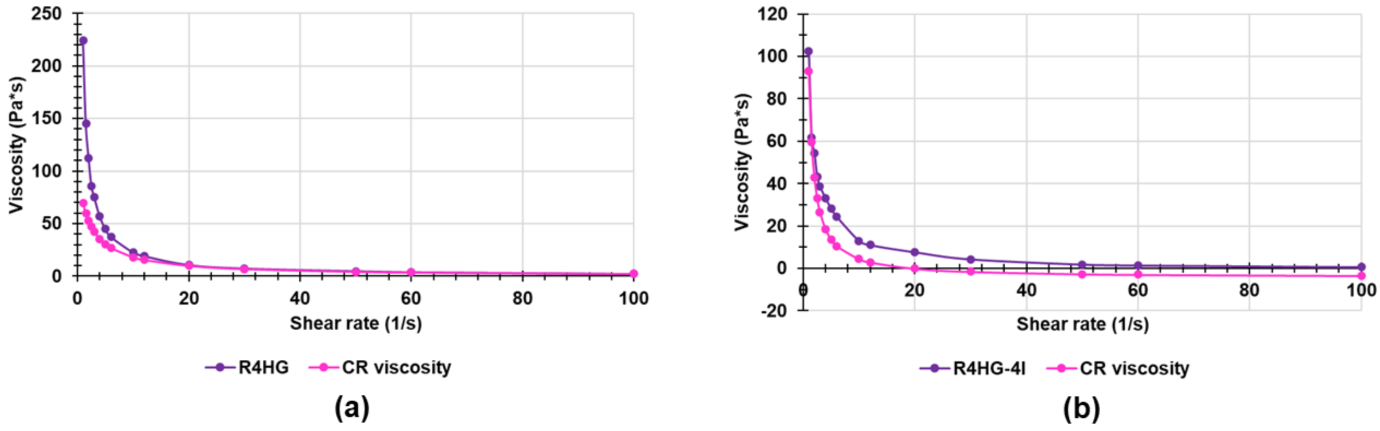

Figure S17. Relationships between the CR viscosity and γ for R4HG (**a**) and R4HG-4I (**b**) (fuchsia lines) and the experimental measurements (purple lines).

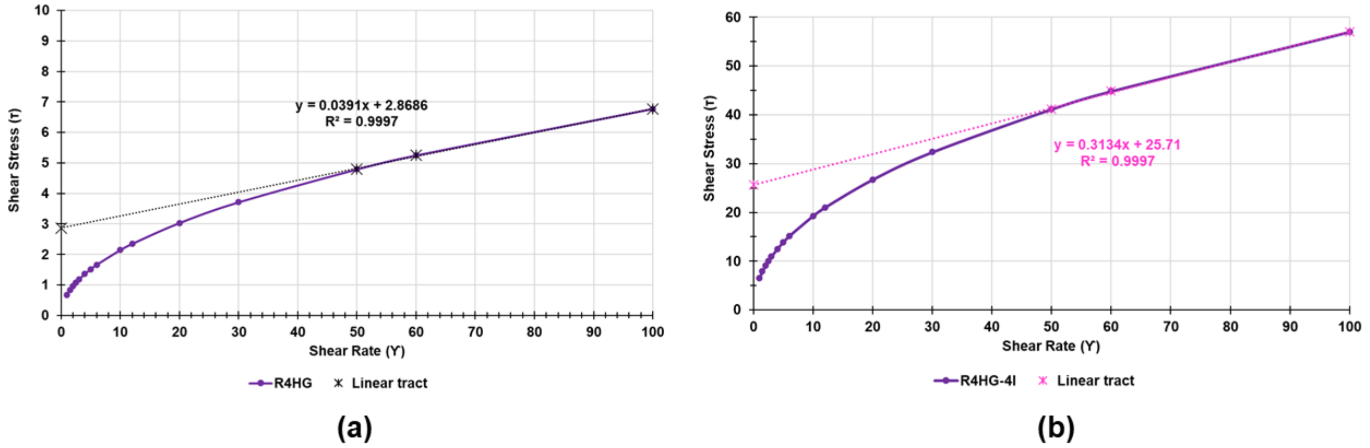

Figure S18. Plots of τ (Pa) vs. γ (s^{-1}) of R4HG (**a**) and R4HG-4I (**b**). The intercept of equations associated with the linear tract of the curves provides the values of the yield stress reported in Table 7 (main text).

Table S2. Values of coefficients of determinations (R^2) obtained for all kinetic models considered.

	Coefficients of Determination (R^2)	
**Kinetic Model**	**R4HG**	**R4HG-4I**
PFO	0.4017	0.4279
**PSO**	**0.9944**	**0.9859**
IPD	0.8354	0.3967

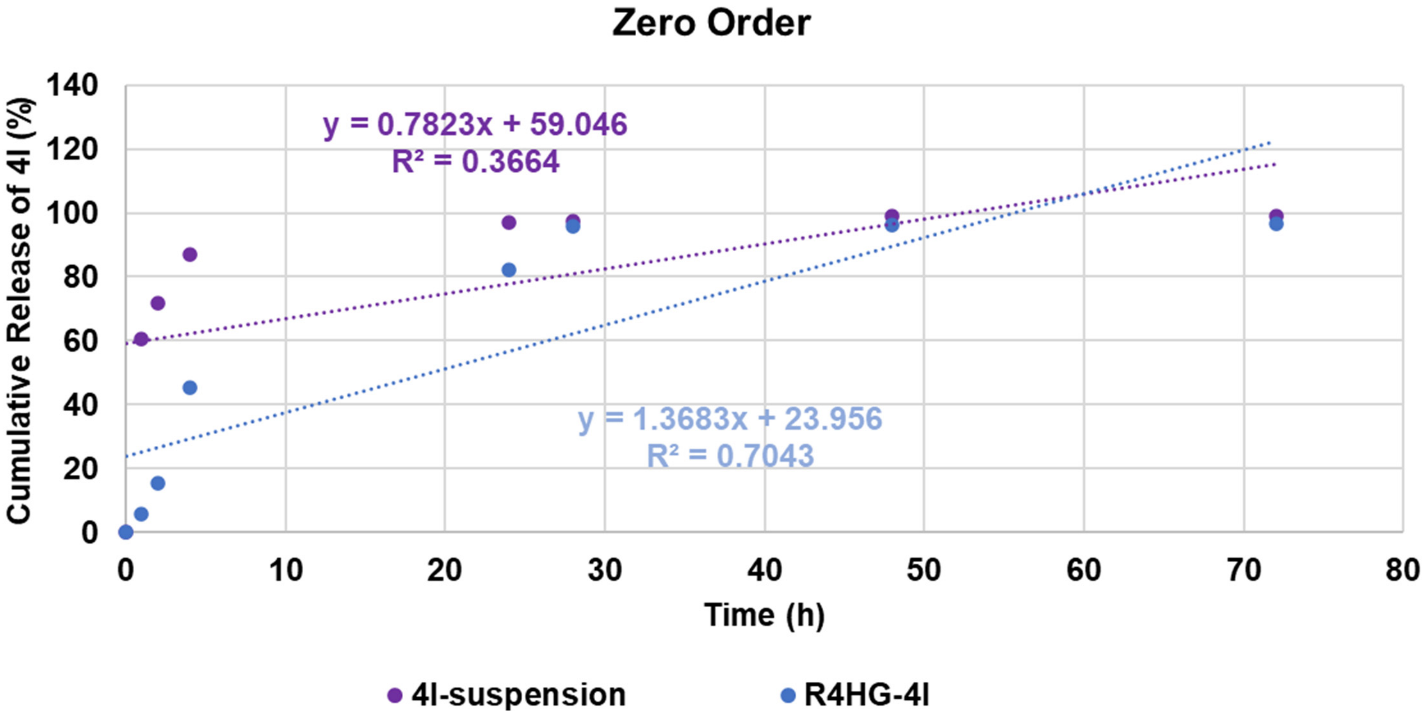

Figure S19. Zero order kinetic models.

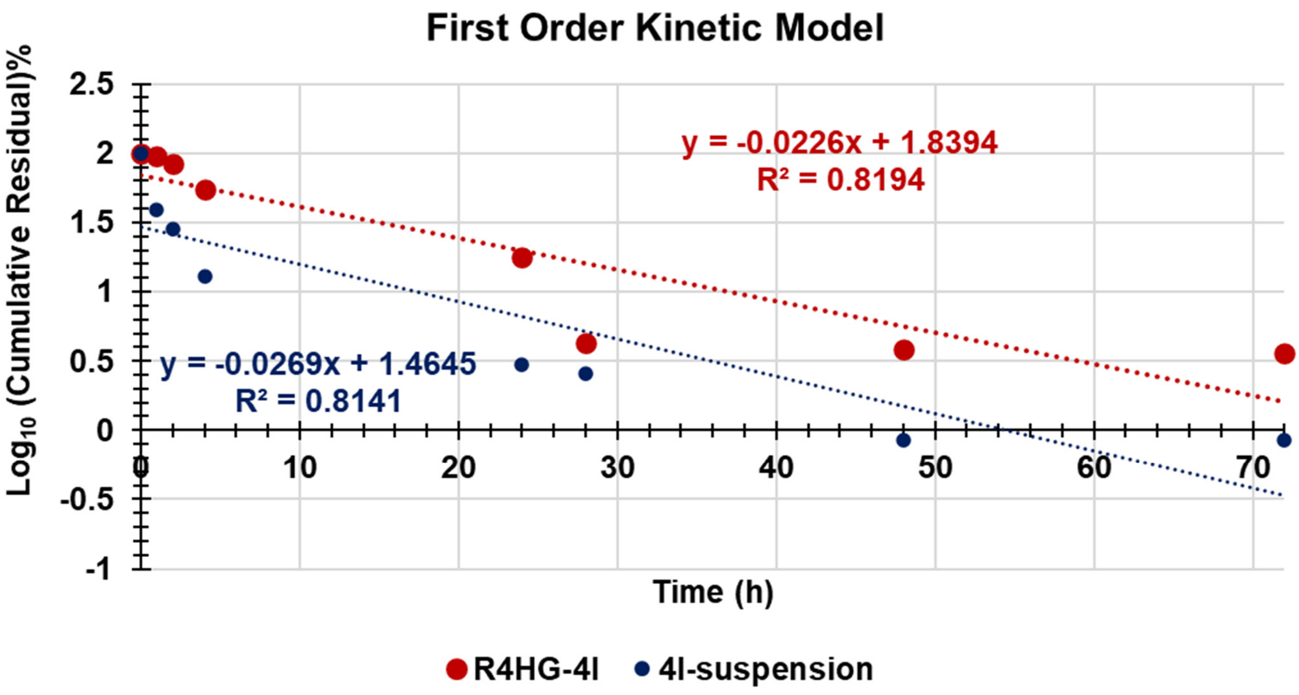

Figure S20. First order kinetic models.

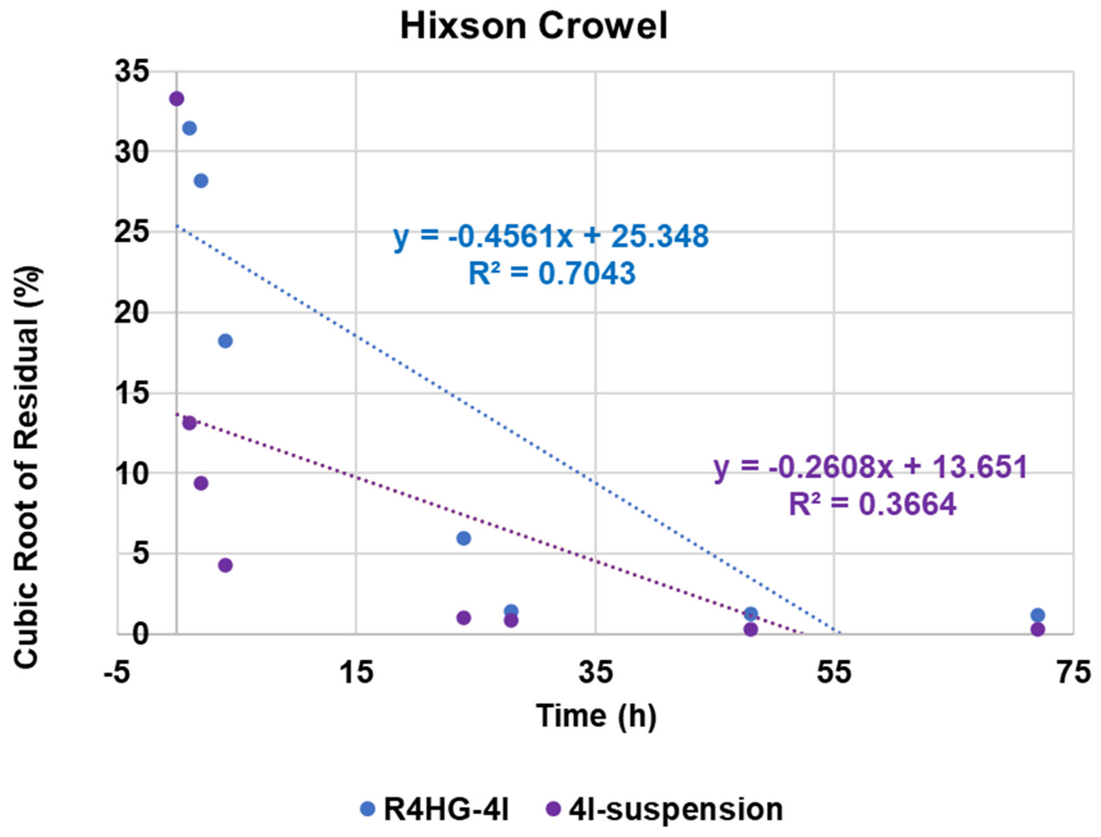

Figure S21. Hixson Crowel kinetic models.

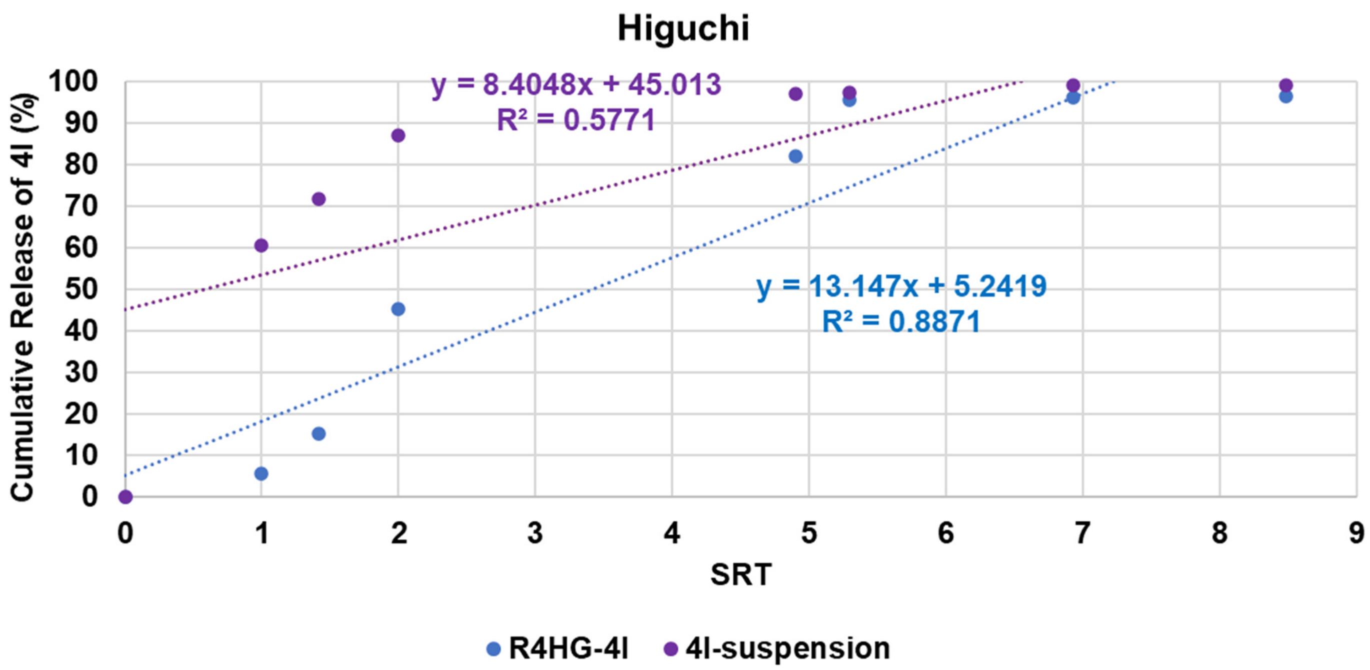

Figure S22. Higuchi kinetic models. SRT = square root of time.

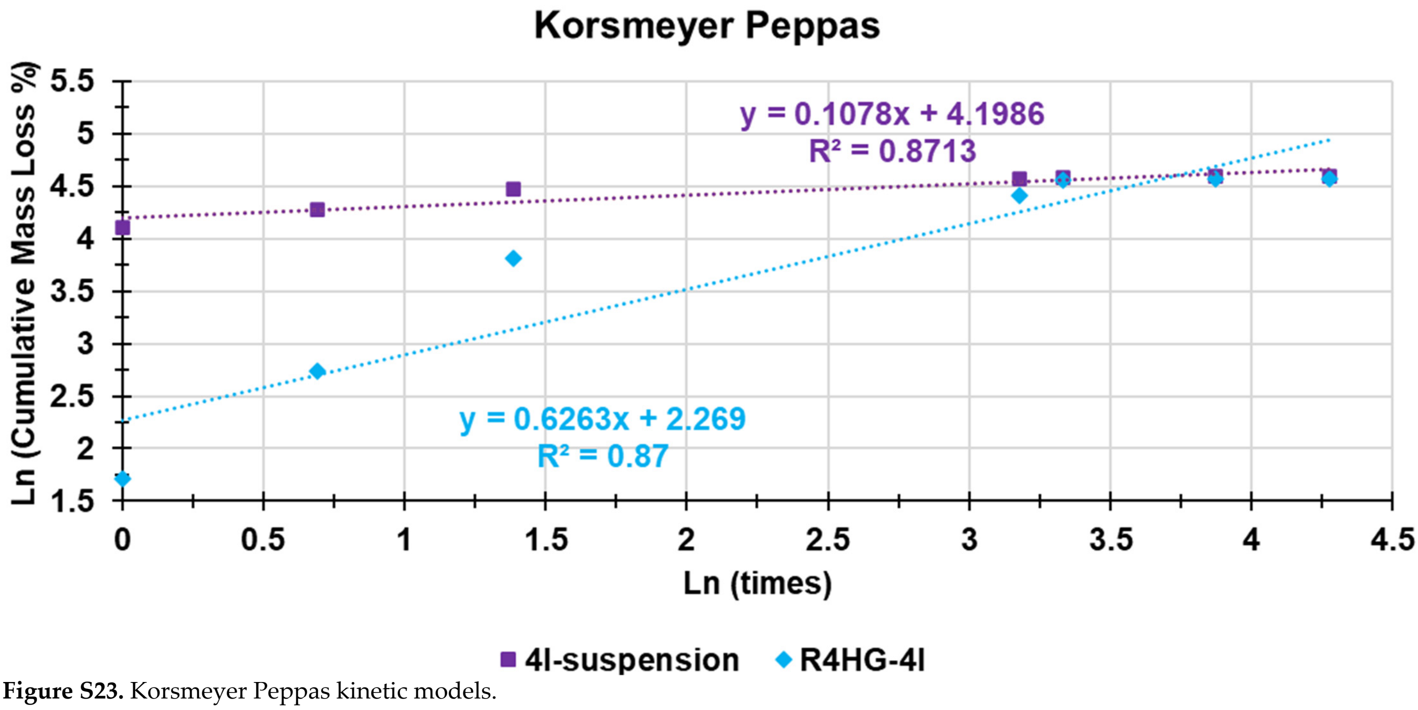

Figure S23. Korsmeyer Peppas kinetic models.

Table S3. Values of the coefficients of determination obtained for all the kinetic models used.

Kinetic Model	R^2 of R4HG-4I	R^2 of 4I-suspension
Zero-order	0.7043	0.3664
First-order	0.8194	0.8141
Hixson–Crowel	0.7043	0.3664
**Higuchi**	**0.8871**	0.5771
**Korsmeyer–Peppas**	0.8700	**0.8713**

Disclaimer/Publisher's Note: The statements, opinions and data contained in all publications are solely those of the individual author(s) and contributor(s) and not of MDPI and/or the editor(s). MDPI and/or the editor(s) disclaim responsibility for any injury to people or property resulting from any ideas, methods, instructions or products referred to in the content.
